# Sodium channel slow inactivation interferes with open channel block

**DOI:** 10.1038/srep25974

**Published:** 2016-05-13

**Authors:** Martin Hampl, Esther Eberhardt, Andrias O. O’Reilly, Angelika Lampert

**Affiliations:** 1 Institute of Physiology and Pathophysiology Friedrich-Alexander Universität Erlangen-Nürnberg, Universitaetsstrasse 17, 91054 Erlangen, Germany; 2 Institute of Physiology, RWTH Aachen University, Pauwelsstrasse 30, 52074 Aachen, Germany; 3 Department of Anesthesiology, Universitaetsklinikum Erlangen, Friedrich-Alexander-Universität Erlangen-Nürnberg, Krankenhausstrasse 12, 91054 Erlangen; 4 School of Natural Sciences and Psychology, Liverpool John Moores University, Liverpool, UK

## Abstract

Mutations in the voltage-gated sodium channel Nav1.7 are linked to inherited pain syndromes such as erythromelalgia (IEM) and paroxysmal extreme pain disorder (PEPD). PEPD mutations impair Nav1.7 fast inactivation and increase persistent currents. PEPD mutations also increase resurgent currents, which involve the voltage-dependent release of an open channel blocker. In contrast, IEM mutations, whenever tested, leave resurgent currents unchanged. Accordingly, the IEM deletion mutation L955 (ΔL955) fails to produce resurgent currents despite enhanced persistent currents, which have hitherto been considered a prerequisite for resurgent currents. Additionally, ΔL955 exhibits a prominent enhancement of slow inactivation (SI). We introduced mutations into Nav1.7 and Nav1.6 that either enhance or impair SI in order to investigate their effects on resurgent currents. Our results show that enhanced SI is accompanied by impaired resurgent currents, which suggests that SI may interfere with open-channel block.

Voltage-gated sodium channels (Navs) are responsible for the fast upstroke and propagation of action potentials in excitable tissues. Channel mutations are linked to inherited diseases such as chronic pain syndromes or epilepsy. The Nav1.7 subtype is predominantly expressed in sensory dorsal root ganglion neurons (DRGs) and mutations leading to hyperexcitability cause the severe neuropathic pain syndromes inherited erythromelalgia (IEM), paroxysmal extreme pain disorder (PEPD) and small-fiber neuropathy (SFN)^1^. In contrast, Nav1.7 loss-of-function mutations produce congenital insensitivity to pain (CIP). IEM and PEPD are both characterized by pain attacks but differ in the distribution of affected body parts, trigger factors, onset of disease during lifetime and autonomic dysfunctions. So far mutagenesis studies have linked both diseases to apparently different pathomechanisms: most IEM mutations discovered to date show a significant hyperpolarizing shift in the voltage dependence of activation whereas all characterized PEPD mutations induce a depolarizing shift of steady-state fast inactivation. However, some recently described IEM mutations evoke hyperexcitability by a depolarizing shift of fast inactivation^2^,^3^. This is surprising as impairment of fast inactivation was believed to be the determining factor for PEPD^4^. Moreover an ‘overlap’ mutation, A1632E, that causes both IEM and PEPD has further challenged the correlation between channel gating and clinical picture, allowing us to develop a more nuanced understanding of this relationship^2^,^5^.

Fast inactivation involves binding of the linker between channel domain III-IV (inactivation particle) to occlude the cytoplasmic end of the pore. Competitive binding to the open pore of an endogenous blocking particle can also terminate ion conductance; the C-terminus of the β4-subunit is the most likely candidate for such an endogenous blocking particle^6^. Unlike recovery from fast inactivation, which involves transition of the pore from the open to closed state^7^, the endogenous blocker can be expelled from the open-state pore during repolarization. This voltage-dependent release of open channel block can generate a resurgent current during the falling phase of an action potential.

All PEPD mutations tested so far show enhanced resurgent currents^1^ whereas all IEM mutations tested to date did not increase resurgent currents. This difference together with altered persistent currents has been proposed to be an electrophysiological basis for the distinct clinical picture of these two diseases^8^. It is not well understood why PEPD mutations enhance resurgent currents and IEM mutations do not. Generally, PEPD mutations also slow the current decay due to fast inactivation, and produce larger persistent currents. These persistent currents have been assumed to support resurgent currents^9^. A characteristic of many IEM mutations is an enhanced slow inactivation (SI).

Here we set out to investigate the effect of enhanced SI on the formation or enhancement of resurgent currents in Navs, thereby furthering our understanding of mutation-induced clinical symptoms (e.g. IEM or PEPD^1^). We selectively enhanced or inhibited SI by introducing residue substitutions into Nav1.7 and also the Nav1.6 channel, which has a distinctly different biophysical profile. We demonstrate for both subtypes that there is a correlation between enhanced SI and reduced resurgent currents, independent of the amount of persistent currents. Our results suggest a new regulatory mechanism for resurgent currents, namely that SI interferes with open channel block.

## Results

### The erythromelalgia mutation Nav1.7/ΔL955 fails to produce resurgent currents despite enhanced persistent currents

**Figure 1:**
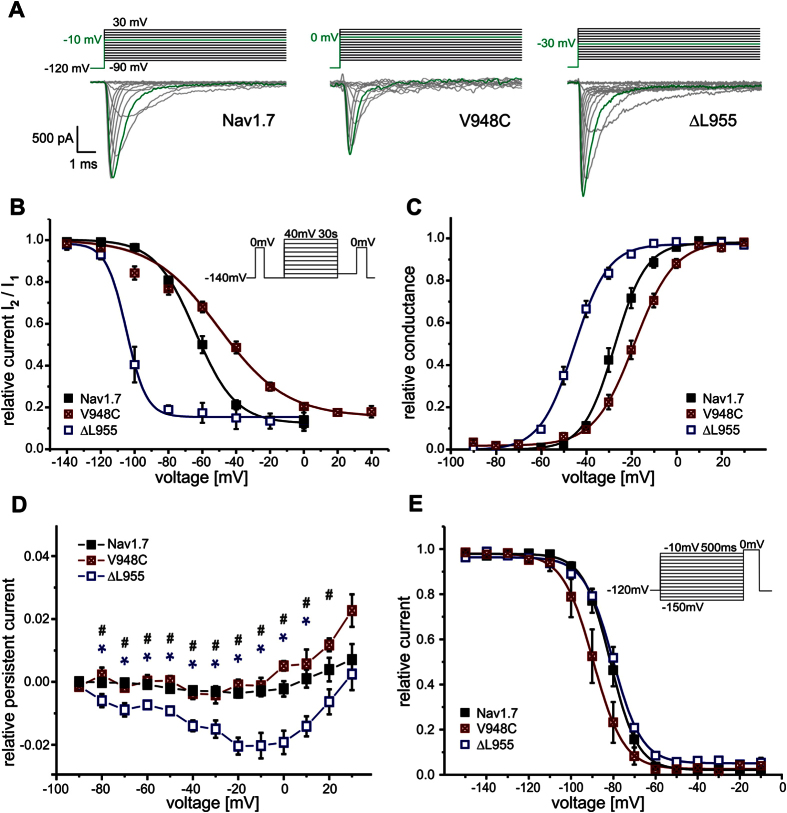
Electrophysiological properties of Nav 1.7 WT and the Nav 1.7/V948C and IEM-linked Nav 1.7/ΔL955 mutant. (**A**) Representative current traces evoked by the indicated voltage protocol in transfected HEK293 cells. For each channel the potential resulting in maximal inward current is colored green. (**B**) Voltage-dependence of steady-state SI shows a −41.9 mV shift of V_1/2_ for Nav1.7/ΔL955 (blue squares, n = 6) to more hyperpolarized potentials and a 16.2 mV shift for Nav1.7/V948C (crossed red squares, n = 6) to more depolarized potentials compared to WT (filled black squares, V_1/2_ = −63.5 ± 2.1 mV, n = 10). (**C**) Normalized conductance–voltage relationships show a −17.7 mV shift of V_1/2_ for Nav1.7/ΔL955 (blue squares, n = 7) to more hyperpolarized potentials and a +8.1 mV shift of V_1/2_ for Nav1.7/V948C (crossed red squares, n = 7) to more depolarized potentials compared to WT (filled black squares, V_1/2_ = −26.8 ± 1.6 mV, n = 6). (**D**) The IEM mutant Nav1.7/ΔL955 shows increased relative persistent currents (n = 14, blue squares) compared to Nav1.7/V948C (crossed red squares, n = 10) and Nav1.7 WT (filled black squares, n = 19). Data shown in (**D**) were recorded in the presence of β4-peptide and 50 nM ATX-II and were significantly different for WT and Nav1.7/ΔL955 (blue*), and for Nav1.7/ΔL955 and Nav1.7/V948C (black^#^), p < 0.05. (**E**) V_1/2_ of steady-state fast inactivation is not altered by Nav1.7/ΔL955 (blue squares, n = 7) whereas Nav1.7/V948C (crossed red squares, n = 7) shifts V_1/2_ by −7.8 mV to more hyperpolarized potentials compared to WT (filled black squares, V_1/2_ = −81.4 ± 1.9 mV, n = 6).

**Figure 2:**
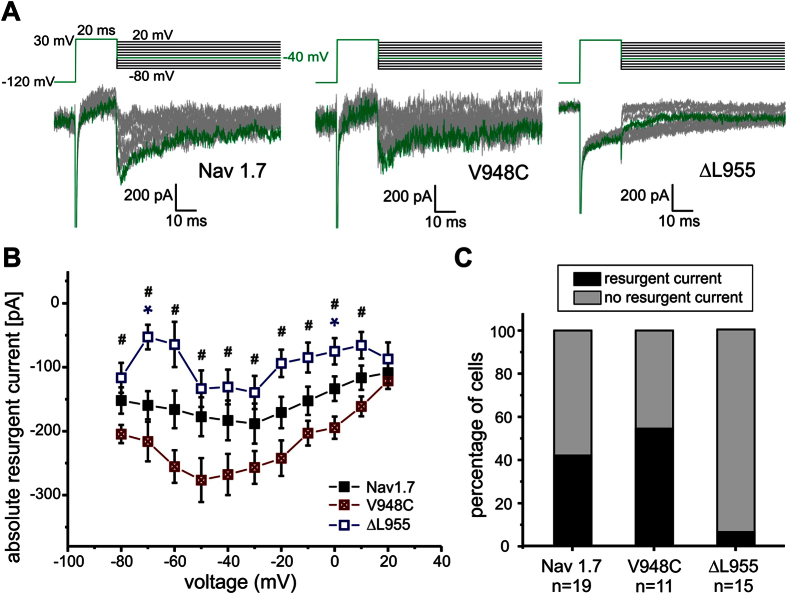
Enhanced SI in Nav1.7 correlates with reduced resurgent currents. (**A**) Representative resurgent current traces recorded by the depicted protocol for Nav1.7 (left panel), the mutation Nav1.7/V948C (middle panel) or the IEM mutation Nav1.7/ΔL955 (right panel) under conditions that favor resurgent currents. The maximal inward resurgent current obtained by a repolarizing pulse to −40 mV is colored green. The mutation Nav1.7/ΔL955 shows smaller resurgent current amplitude (**B**) and less frequent occurrence of resurgent currents (**C**) whereas in the Nav1.7/V948C mutation and Nav1.7 WT resurgent currents could be evoked in 55% and 42% of measured cells, respectively (black filled bars). Significant differences between Nav1.7 WT and Nav1.7/ΔL955 are marked with blue* and between Nav1.7/ΔL955 and Nav1.7/V948C with ^#^p < 0.05.

Many IEM mutations display enhanced SI but none of these show resurgent currents^1^,^8^. SI of the IEM mutation Nav1.7/ΔL955 is strongly enhanced (Fig. 1A,B). In addition to the pronounced hyperpolarizing shift of the midpoint (V_1/2_) of activation (Fig. 1C, −26.8 ± 1.6 mV for wildtype (WT) and −44.5 ± 1.3 mV for Nav1.7/ΔL955, ΔV_1/2_ = −17.7 mV), Nav1.7/ΔL955 produces enhanced persistent currents (Fig. 1D). In addition to persistent currents, Nav1.7/ΔL955 was reported to have a slower current decay^10^, a combination that would predispose it to generate resurgent currents. We therefore tested this mutation under conditions that would enhance any occurring open channel block. HEK cells lack the endogenous blocker and therefore resurgent currents are normally absent (Fig. S1). In an experimental setting with 100 μM β4-peptide in the pipette and 50 nM ATX-II in the bath solution as described previously^11^ (see Methods section) we observed resurgent currents in Nav1.7 WT (Fig. 2A,B) in about 50% of the tested cells (Fig. 2C). Despite optimizing all conditions to generate resurgent currents only one out of 15 cells expressing the Nav1.7/ΔL955 mutation displayed resurgent currents, which were very small (Fig. 2). This clearly contrasts with the enhanced persistent currents that we (Fig. 1D) and others^10^ described for the Nav1.7/ΔL955 mutation.

### Nav1.7/V948C impairs SI but enhances resurgent currents

As SI is negatively shifted in the Nav1.7/ΔL955 mutation and resulted in diminished resurgent currents, we questioned whether a depolarizing shift in SI would have the opposite effect on the generation of resurgent currents. For this reason we mutated residues in the pore region of Nav1.7 that were shown to affect SI of Nav1.4. In the Nav1.7/V948C mutation (corresponding to Nav1.4/V787C^12^) SI is strongly reduced with a depolarizing shift of the midpoint (V_1/2_) of SI of +16.2 mV (Fig. 1B, −63.5 mV ± 2.1 mV for WT and −47.3 mV ± 2.8 mV for V948C) whereas activation and steady-state fast inactivation were affected to a much lesser extent (activation ΔV_1/2_ = +8.1 mV, steady-state fast inactivation ΔV_1/2_ = −7.8 mV, Fig. 1C,E, supplementary Table S1). Surprisingly Nav1.7/V948C displays no persistent currents, which would typically be considered to render the channel unlikely to produce resurgent currents (Fig. 1D).

Under conditions that would support open channel block, the SI deficient Nav1.7/V948C mutation still showed no persistent currents, whereas its resurgent currents were clearly detectable in a substantial subset of patched cells, especially when compared to Nav1.7 WT (Fig. 2). Six out of eleven cells (55%) showed resurgent currents for the Nav1.7/V948C mutation, whereas in Nav1.7 WT transfected cells only eight out of 19 cells (42%) displayed this current.

These findings suggest a negative correlation between SI and resurgent currents, as the SI-reducing Nav1.7/V948C mutation displays clear resurgent currents, whereas the Nav1.7/ΔL955 mutation, with strong SI, showed almost no resurgent currents (Fig. 2B,C, Table 1).

### Subtype Specificity: SI of Nav1.7 WT is more pronounced than that of Nav1.6 WT

The pain-linked Nav1.7 WT channel produces little-to-no resurgent currents^13^. In contrast, Nav1.6 is believed to be the predominant carrier of resurgent currents in Purkinje cells^14^ and also DRGs^15^. Interestingly Nav1.6 is described as relatively resistant to SI^16^ and both features promote high-frequency firing of action potentials.

**Figure 3:**
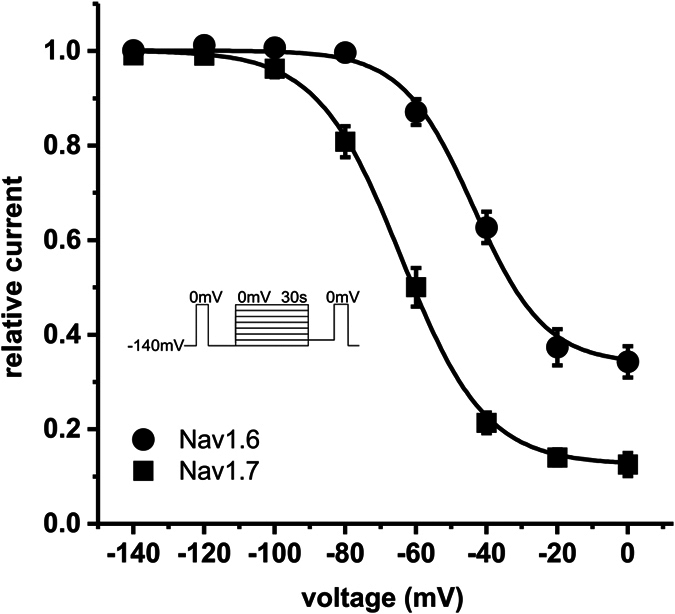
SI is more pronounced in Nav1.7 WT than in Nav1.6 WT. SI of Nav1.7 WT develops at more than 20 mV more hyperpolarized potentials than SI of Nav1.6 WT (V_1/2_ = −63.5 ± 2.1 mV for Nav1.7 (filled black squares, n = 8) and −43.4 ± 1.2 mV for Nav1.6 WT (filled black circles, n = 7). Note that maximum amount of SI at depolarized potentials is also different (90.0% for Nav1.7 WT and 66.9% for Nav1.6 WT). For the data shown in this figure the identical protocol was used for Nav1.7 and for Nav1.6 (see inset).

The voltage-dependence of steady-state SI is more than 20 mV more hyperpolarized in Nav1.7 (V_1/2_ = −63.5 ± 2.1 mV, n = 10) compared to Nav1.6 (V_1/2_ = −43.4 ± 1.2 mV, n = 7) (Fig. 3). The maximum amount of channels undergoing SI revealed by Boltzmann fits was significantly different for the two channels (90.0% for Nav1.7 and 66.9% for Nav1.6, p < 0.001). Even an increased prepulse length of 60 s did not enhance SI of Nav1.6 to the extent observed in Nav1.7 (supplemental Table S1). As our data on Nav1.7 suggest that strong SI may impair the formation of resurgent currents, we hypothesized that a mutation that enhances SI in Nav1.6 may decrease or abolish resurgent currents.

### The pore mutations Nav1.6/V966C and Nav1.6/N1455A strongly shift steady-state SI in opposite directions

The clinically-relevant ΔL955 mutation has to-date only been described in Nav1.7 whereas an asparagine mutation in the pore has been described in two channel isotypes: Nav1.2^17^ and Nav1.4^18^ and found to strongly affect SI without altering other biophysical properties of the corresponding channel. Therefore we generated the equivalent mutation (N1455A) in Nav1.6 to study enhanced SI in this channel. We generated a second pore mutant of Nav1.6: Nav1.6/V966C, which is the equivalent mutation to Nav1.7/V948C and therefore predicted to have impaired SI.

**Figure 4:**
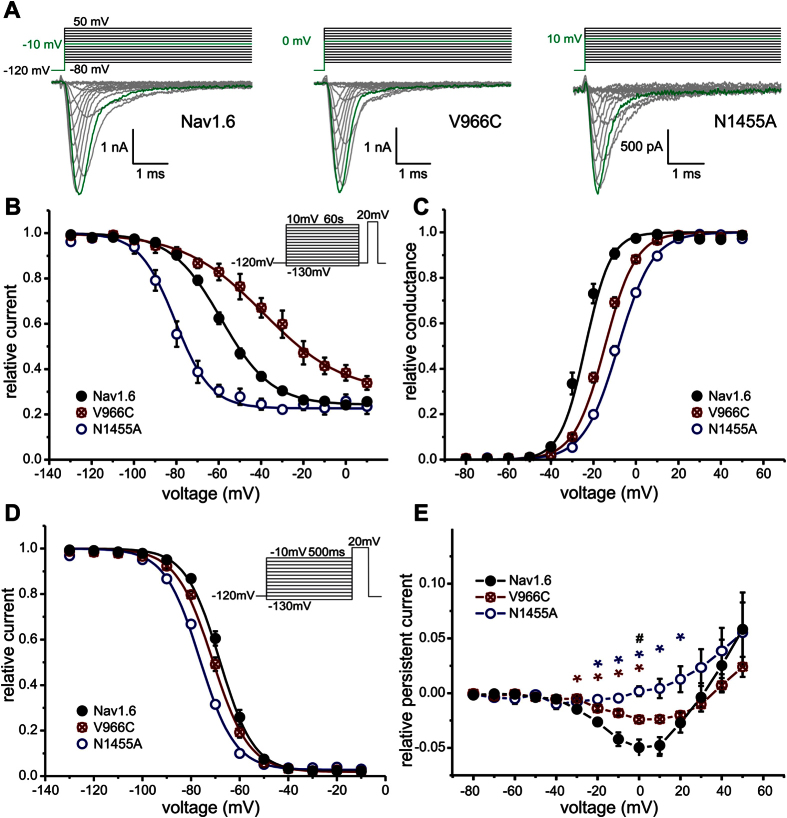
SI of Nav 1.6 is modified by the mutations Nav1.6/V966C and Nav1.6/N1455A. (**A**) Representative current traces of Nav1.6 (left panel), the mutation Nav1.6/V966C (middle panel) and the mutation Nav1.6/N1455A (right panel) expressed in ND7-cells. For each channel the maximal inward current and corresponding potential is colored in green. (**B**) Steady-state SI is enhanced by the Nav1.6/N1455A mutation (open blue circles, V_1/2_ = −81.9 ± 2.5 mV, n = 4) and decreased by the Nav1.6/V966C mutation (crossed red circles, V_1/2_ = −33.8 ± 5.1 mV, n = 5) compared to Nav1.6 WT (filled black circles, V_1/2_ = −59.6 ± 1.5 mV, n = 13). (**C**) Normalized conductance–voltage relationship is shifted to more depolarized potentials for both Nav1.6 mutants whereas (**D**) the V_1/2_ of steady-state fast inactivation is slightly hyperpolarized. (**E**) Nav1.6 WT (filled black circles) and the mutant Nav1.6/V996C (crossed red circles) display persistent currents but the mutant Nav1.6/N1455A (open blue circles) does not. Data shown in E were recorded in the presence of β4-peptide and 50 nM ATX-II and were significantly different for Nav1.6 WT and Nav1.6/N1455A (blue*), for Nav1.6 WT and Nav1.6/V966C (red*) and for Nav1.6/N1455A and Nav1.6/V966C (black^#^), p < 0.05.

Nav1.6/V966C strongly shifts voltage-dependence of SI to more depolarized potentials by +25.8 mV (V_1/2_ = −59.6 mV ± 1.5 mV for WT and V_1/2_ = −33.8 mV ± 5.1 mV for V966C, Fig. 4B). Nav1.6/N1455A has the opposite effect: it hyperpolarizes V_1/2_ by −22.3 mV (Fig. 4B, V_1/2_ = −81.9 mV ± 2.5 mV for N1455A). Boltzmann fits revealed that the maximal relative block due to SI in both mutants was not significantly different compared to Nav1.6 WT (supplemental Table S2). Both mutations shifted activation to more depolarized potentials (ΔV_1/2_ = 9.9 mV and 16.7 mV for V966C and N1445A, respectively, Fig. 4C) and voltage-dependence of steady-state fast inactivation was hyperpolarized (ΔV_1/2_ = −3.1 mV and −8.6 mV for V966C and N1455A, respectively, Fig. 4D, supplemental Table S1).

Thus, mutations in the pore of Nav1.6 are capable of strongly enhancing (Nav1.6/N1455A) or reducing (Nav1.6/V966C) SI with only mild effects on activation or steady-state fast inactivation.

### Nav1.6 WT and Nav1.6/V966C display persistent currents and large resurgent currents

Under conditions that favor open channel block, Nav1.6 WT and the V966C mutation displayed small persistent currents. This contrasts with the corresponding V948C substitution in Nav1.7 that did not show any persistent currents (see Fig. 1D). Persistent currents were completely abolished in the Nav1.6/N1455A mutation (Fig. 4E).

**Figure 5:**
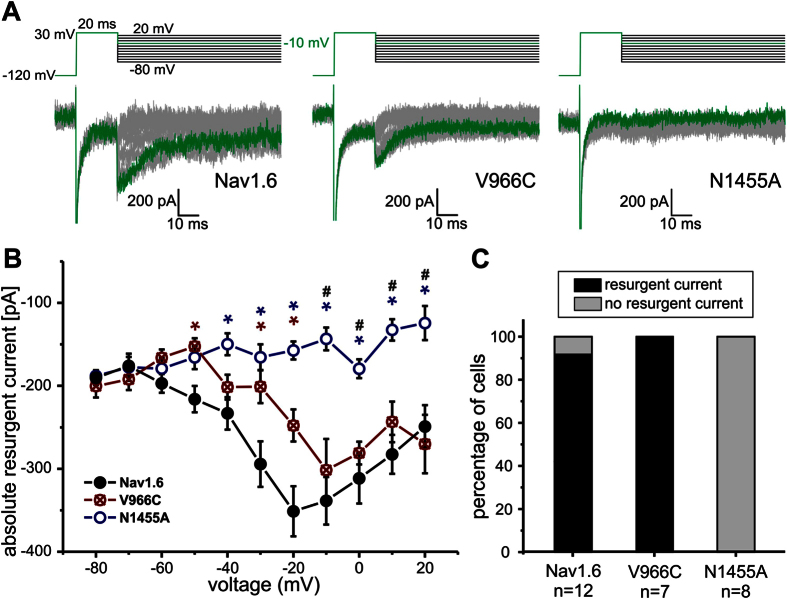
Enhanced SI in Nav1.6 correlates with reduced resurgent currents. (**A**) Representative resurgent current traces recorded by the depicted protocol of Nav1.6 (left panel), the mutation Nav1.6/V966C (middle panel) and the mutation Nav1.6/N1455A (right panel) under conditions that favor resurgent currents. The maximal resurgent current obtained by a repolarizing pulse to −10 mV is colored green. Cells expressing Nav1.6 WT and the Nav1.6/V966C mutant show robust resurgent currents (**B**) in 90 to 100% of cells (black bars) (**C**) whereas the N1455A mutant was not able to produce any resurgent currents. Significant differences between Nav1.6 WT and Nav1.6/N1455A are marked with blue*, between Nav1.6 WT and Nav1.6/V966C with red* and between Nav1.6/N1455A and Nav1.6/V966C with ^#^p < 0.05.

The SI-deficient Nav1.6/V966C mutation displayed clearly detectable resurgent currents in 100% of the tested cells whereas the Nav1.6/N1455A mutation, which had a strongly enhanced SI, did not produce any detectable resurgent currents (Fig. 5C). Enhancement of SI seems to have abolished resurgent currents. Thus, in Nav1.6 as well as in Nav1.7 reduced SI may lead to enhanced resurgent currents, whereas persistent currents do not seem to be necessarily required (summarized in Table 1).

Our results suggest that SI in Nav1.7 as well as in Nav1.6 interferes with open channel block and thus may present a new mechanism to suppress the generation of resurgent currents.

### Lacosamide enhances SI and reduces resurgent currents

**Figure 6:**
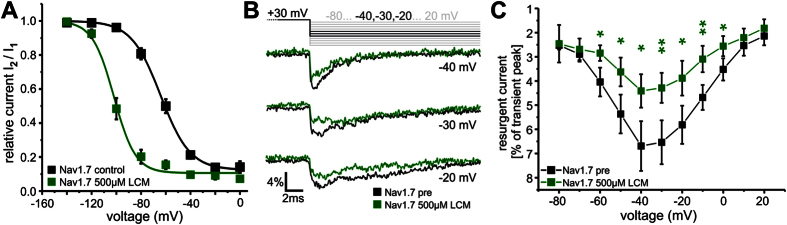
LCM enhances SI and reduces resurgent currents. (**A**) 500 μM LCM shift voltage-dependence of steady-state SI of Nav1.7 WT to more hyperpolarized potentials (ΔV_1/2_ = 36.3 mV, V_1/2_ = −63.5 ± 2.1 mV for Nav1.7 control (filled black squares, n = 10) and −99.8 ± 1.2 mV for Nav1.7 500 μM LCM (filled green squares, n = 6)). The data were recorded with the protocol depicted in Fig. 3 (inset). (**B**) Representative resurgent currents traces of Nav1.7 recorded at the indicated test pulses before (black) and after application of 500μM LCM (green) via perfusion to the same cell. (**C**) Resurgent current amplitude (black squares) decreased after the application of 500 μM LCM (filled green squares, n = 6). Transient peak current was reduced by LCM, therefore resurgent currents are displayed as percentage of transient peak currents of each trace and plotted as a function of voltage. *p < 0.05 and **p < 0.005 (paired t-test).

Lacosamide (LCM) is a known Nav1.7 modulator in clinical use as an antiepileptic. Consistent with previous reports, LCM strongly enhances SI of Nav1.7 (Fig. 6A) with little effect on activation or fast inactivation (Fig. S2A,^19^). Upon application of 500 μM LCM resurgent currents in WT Nav1.7 were significantly reduced (Fig. 6B,C and Fig. S2B), supporting our hypothesis that selective enhancement of SI impairs resurgent current formation.

## Discussion

Our studies disclose a relationship between SI and resurgent currents. Nav1.7 and Nav1.6 differ considerably in their propensity for SI and resurgent currents and we mutated both channels to further study these and other biophysical properties (Table 1). Nav1.7/ΔL955 and Nav1.6/N1455A enhanced SI but lead to an attenuated capability to produce resurgent currents. In contrast Nav1.7/V948C and Nav1.6/V966C mutations impaired SI but generated larger resurgent currents. Thus, there appears to be a negative correlation between the extent of SI and the capability to generate resurgent currents.

The current mechanism proposed for resurgent currents is that an endogenous blocker – most likely the positively-charged C-terminus of the Nav β4 subunit^6^,^20^,^21^ – binds to and blocks the open pore of an activated Nav channel. Upon repolarization the blocker unbinds to allow current flow, thus generating resurgent currents. Fast inactivation and resurgent currents have been described as mutually exclusive as binding of the fast inactivation particle on the DIII-DIV linker precludes binding of the resurgent-current blocker^22^. Instead, resurgent currents have to date been considered strongly associated with persistent currents.

Besides the so-called “window” current observed at voltages where channels activate but inactivation is incomplete, persistent current is mainly explained by two hypotheses: (1) channels may have an incomplete equilibrium occupancy of inactivated states^23^, and (2) persistent current is a short lived closed-state distinct from the inactivated state, which occurs when inactivation is slow or does not take place while channels are able to reopen by bursting channel behavior. Probably even more than one “persistent current state” exists^24^,^25^. Bant and Raman^20^ also hypothesize that the blocking particle could be involved in both currents: Persistent currents could occur by rapid binding and unbinding with low affinity at negative voltages and resurgent currents require strong de- and repolarizations to expel the blocking particle bound with high affinity that inhibits persistent currents.

It seems intuitive that an open pore that conducts persistent currents would also increase the probability for the blocking particle to bind, thus producing more pronounced resurgent currents^6^,^8^,^26^,^27^ and indeed our data for the Nav1.6/N1455A mutation fit with this model. However, this does not hold true for the other mutations we investigated. Despite pronounced persistent currents in the IEM mutation Nav1.7/ΔL955^10^, no resurgent currents were observed. Surprisingly, the Nav1.7/V948C mutation with no persistent currents exhibited increased resurgent currents. Furthermore, all cells expressing Nav1.6/V966C displayed more resurgent currents compared to WT, even though persistent currents were twice the size in Nav1.6/WT compared with Nav1.6/V966C.

It is conspicuous that the voltage range in which persistent and resurgent currents arise is largely the same for both channel isoforms and their mutants tested here. Therefore one can assume that both current states can arise at the same voltage and thus might be alternative gating modes that both become more likely if for example fast inactivation is slow. Interestingly, there was a strong negative correlation between SI and resurgent currents that has also been described for persistent currents^24^. SI could therefore represent another gating mode competing with short lived closed states. Based on the knowledge we have up to now on persistent and resurgent currents our findings suggest that persistent currents and resurgent currents could both resemble two competing short-lived closed states that might be two alternatives that can be favored when inactivation is slowed and SI did not yet occur.

**Figure 7:**
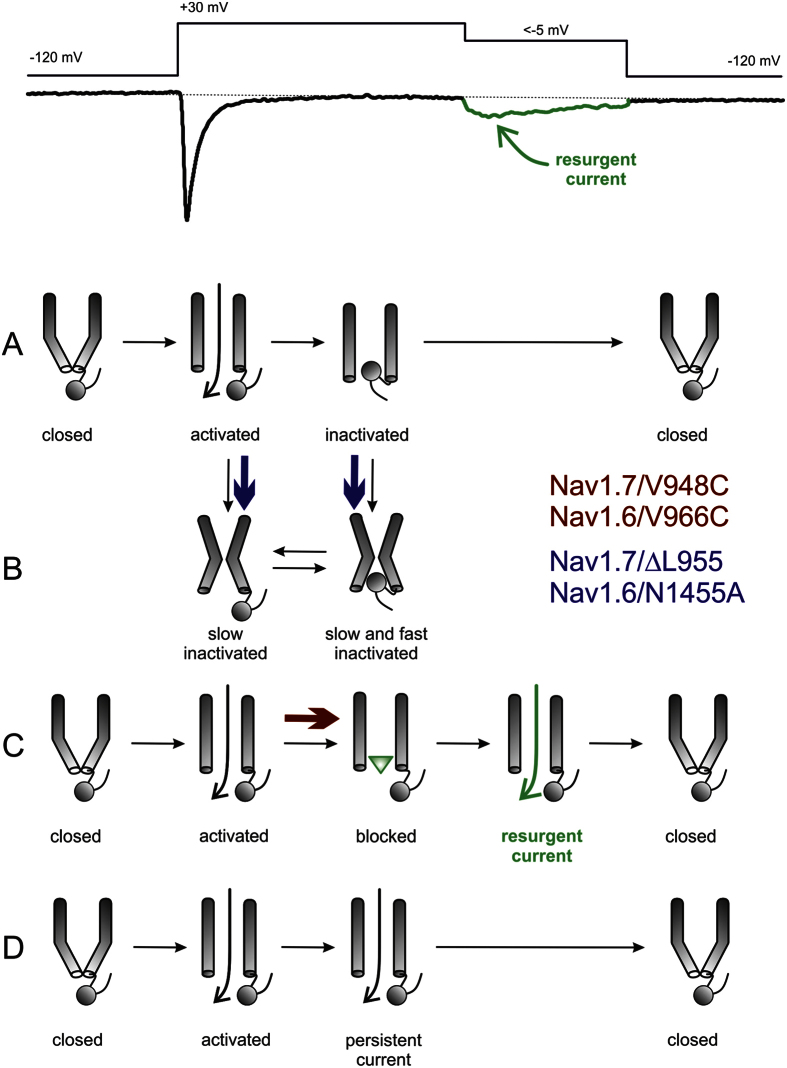
Schematic display of gating transitions of Navs during a resurgent current protocol (shown on top with an exemplary resurgent current recording). (**A**) Following activation the channel undergoes fast inactivation via the inactivation particle. (**B**) From both the open and fast inactivated state, the channel may undergo SI. (**C**) In the presence of an open channel blocker (green triangle) the channel may be blocked. Dissociation of the blocker during membrane repolarization gives rise to a resurgent current. (**D**) Some channels may not undergo fast inactivation nor open channel block, and produce persistent currents. Additional states and transitions between depicted states are likely to occur, and are not captured in this simple schematic overview. Blue arrows show the transitions that are facilitated by the SI enhancing mutations (Nav1.7/ΔL955, Nav1.6/N1455A), whereas the red arrow indicates the facilitated block by the open channel blocker (green triangle, Nav1.7/V948C, Nav1.6/V966C), leading to resurgent currents.

The poor correlation we observed between persistent currents and resurgent currents contrasts with the strong relationship we discovered between SI and resurgent currents. The structural basis underlying SI remains poorly understood but is believed to involve structural rearrangements of the pore, which is mechanistically analogous to C-type inactivation in potassium channels^28^. It is a separate process that does not involve the inactivation particle on the DIII-DIV linker^29^ (Fig. 7). Numerous site-directed mutagenesis studies have identified residues in the S6 segments that affect SI^18^,^30^,^31^,^32^,^33^, supporting the hypothesis that SI involves structural changes and possible collapse of the pore region. The link between SI and the pore has also been demonstrated pharmacologically. Local anesthetics (LA) that bind to their receptor site in the pore can affect SI e.g. lidocaine reduces transition to SI^34^ and stabilizes the SI state^35^.

The binding site for the resurgent-current blocking particle overlaps with the local anesthetic (LA) receptor^20^. We therefore propose that SI-associated structural rearrangements of the pore may disrupt the receptor for the resurgent-current blocking particle or could obstruct access to the pore for this blocker. This could explain the relationship we observed between enhanced SI and attenuated resurgent currents. Accordingly, the impairment of SI could increase the availability of the pore receptor for the blocking particle, leading to greater resurgent currents. While we cannot rule out that mutagenesis might have knocked out a direct binding contact for the resurgent-current blocker, the congruity of our findings across six constructs involving three different mutations and two distinct Nav subtypes indicates that a general correlation exists between the extent of SI in a channel and its capability to generate resurgent currents: a negative shift in the voltage-dependence of SI is associated with decreased resurgent currents.

Most IEM mutations not only enhance SI but also increase its voltage-sensitivity^4^,^10^,^36^,^37^,^38^,^39^,^40^,^41^,^42^,^43^. We also observed a steeper SI-voltage relationship in the Nav1.7/ΔL955 and Nav1.6/N1455A mutants (Figs 1 and 4), which may influence the overall conformation of the pore, rendering it more likely to undergo SI and thus impairing the binding of an open channel blocker.

Resurgent currents have not been found in any IEM-associated mutations of Nav1.7 investigated to date^1^. In light of our current findings, we suggest that the hyperpolarized shift of SI that is common to IEM mutants could account for this absence of resurgent currents. Indeed, this relationship between enhanced SI and absent resurgent currents may be a key determinant of IEM symptoms. For example, in a recent study Emery *et al*.^3^ characterized the Nav1.7/L245V mutation that exhibits depolarized steady-state fast inactivation with no change of activation. Despite large persistent currents that have up-to-now been characteristic of PEPD, the clinical phenotype of the Nav1.7/L245V patient was IEM. In agreement with our results and in support of our hypothesis, the mutant displayed no resurgent currents and SI was enhanced.

Resurgent currents appear to be decisive for the PEPD phenotype, whereas persistent currents may not be as important as previously suggested^2^,^8^,^9^,^27^, especially considering the Nav1.7/ΔL955 and L245V IEM-associated mutations that both exhibit large persistent currents. Every PEPD mutant tested so far has been shown to produce resurgent currents and none has been reported with enhanced SI; in these channels SI remains unmodified or is impaired. Therefore it may be the interplay between SI and resurgent currents that ultimately determines whether the PEPD or IEM phenotype manifests. LCM is an antiepileptic drug in clinical use that enhances SI of Nav1.7^19^. Our experiments show a strong reduction of resurgent currents by this compound, suggesting that using drugs that selectively target SI of Nav1.7 may be helpful in the future to treat patients suffering from PEPD or other forms of pain.

In this study we determined a negative correlation between SI and resurgent currents. Our findings may have clinical relevance, as enhancement of SI by LCM, a drug already in clinical use, reduced resurgent currents and therefore has potential as treatments for PEPD patients. Beside LCM other SI-enhancing drugs are known, such as eslicarbazepine^44^. In general SI-modifying compounds might offer new opportunities in the treatment not only of hereditary pain disorders but for the therapy of sporadic chronic and acute pain.

## Experimental Procedures

### Cell culture and transfection

All channel constructs were generated based on WT hNav1.7 and mNav1.6-tetrodotoxin (TTX)r in modified pcDNA3 vectors and the following mutations were generated using the QuikChange XL site-directed mutagenesis kit (Stratagene): hNav1.7/V948C, hNav1.7/ΔL955, mNav1.6-TTXr/V966C and mNav1.6-TTXr/N1455A. Wildtype or mutant channels were transiently transfected into HEK293 cells for hNav1.7 or neuronal cell lines ND7/23 and N1E115 for mNav1.6 with Nanofectin (PAA Laboratories GmbH) according to the manufacturer’s protocol.

HEK293 cells were maintained in DMEM medium (Gibco-Life technologies) including 10% FBS, 1.0 g/l Glucose and 1% Penicillin/Streptomycin (PAA Laboratories GmbH) whereas neuronal cell lines required 4.5 g/l Glucose. If not indicated otherwise 0.5 μg EGFP-C1 (Clontech Laboratories, Inc., Mountain View, USA) was cotransfected in order to detect transfected cells via green fluorescence. Except for measurement of resurgent currents mNav1.6 and its mutants were coexpressed with a GPF-tagged mouse β4-subunit in a 4:1 ratio to enhance current density. Cells were recorded 1 to 2 days after transfection.

### Electrophysiology

Whole-cell voltage clamp experiments of transfected cells were performed with an EPC-10USB amplifier (HEKA Elektronik, Lambrecht, Germany) at room temperature. Glass pipettes (tip resistance 1.5–2 MΩ) were manufactured with a DMZ puller (Zeitz Instruments GmbH, Martinsried, Germany) and filled with internal solution containing (in mM): 140 CsF, 10 NaCl, 10 Hepes 1 EGTA, 10 Glucose (pH 7.4, adjusted with CsOH). The bath solution contained (in mM): 140 NaCl, 3 KCl, 1 MgCl_2_, 1 CaCl_2_, 10 Hepes, 20 Glucose (pH 7.4, NaOH). For recording of Nav1.6 currents 500 nM TTX (Biotrend AG, Wangen, Switzerland) was added to block endogenous sodium currents.

Capacitive transients were cancelled and series resistances (<5 MΩ) were compensated by at least 65%. Leak currents were subtracted digitally online using the P/4 procedure following the test pulses. Signals were digitized at sampling rates between 20 kHz to 100 kHz adapted to the voltage protocol length and purpose. Voltage protocols were carried out after current stabilization and equilibration was established using a holding potential of −120 mV. For SI measurements of hNav1.7 and its mutations a holding potential of −140 mV was used. Patchmaster/Fitmaster software (HEKA Elektronik, Lamprecht, Germany) was used for acquisition and off-line analysis.

Current-voltage (I–V) relations were obtained using 100 ms pulses to a range of test potentials in 10 mV steps with an interpulse interval of 5 seconds. The voltage-dependent sodium channel conductance G_Na_ was calculated using the following equation: G_Na_ = I_Na_/(V_m_ − E_rev_) where I_Na_ is the amplitude of the current at the voltage V_m_, and E_rev_ is the reversal potential for sodium, which was determined for each cell individually.

Activation curves were derived by plotting normalized G_Na_ as a function of test potential and fitted with the Boltzmann equation: G_Na_ = G_Na, max_/(1 + exp [(V_m_ − V_1/2_)/*k*]) where G_Na, Max_ is the maximum sodium conductance, V_1/2_ is the membrane potential at half-maximal activation, V_m_ is the membrane voltage and *k* is the slope factor.

Persistent sodium currents are given as the mean remaining sodium current between 66 to 99 ms for each test pulse in the presence of 50 nM ATX-II in the bath and 100 μM β4-peptide in the pipette.

Resurgent currents were assessed using a 20 ms depolarizing voltage step to +30 mV to allow channel opening followed by a 500 ms test pulse in steps of 10 mV (ranging from −80 mV to 20 mV) with ATX-II in the bath and the β4-peptide in the internal solution. Peak resurgent currents were analyzed between 0.6 ms and 5 ms of the repolarizing test pulse and only currents showing a slowly activating component which was clearly distinguishable from tail currents were counted as resurgent current positive. Activation traces with the respective voltage step were subtracted from recorded resurgent current traces to minimize the artifact of persistent currents which occurs after the first depolarizing voltage pulse in the resurgent current protocol, especially in the hNav1.7/ΔL955 mutant. The resulting resurgent currents are given in absolute values measured as the peak inward current following the prepulse and are plotted against the voltage of the test pulse.

Voltage-dependence of steady-state fast inactivation was measured using a series of 500 ms prepulses (−130 mV or −150 mV for mNav1.6 or hNav1.7 respectively to −10 mV in each case), followed by a 40 ms supramaximal depolarization to +20 mV (mNav1.6) or 0 mV (hNav1.7) that served as a test pulse to assess the available non-inactivated channels. Normalized peak inward current amplitude (I_Na_/I_Na, Max_) at each test pulse is displayed as a function of prepulse potential (V_m_) and fitted using the following (Boltzmann) equation I_Na_/I_Na, max_(V) = 1/(1+ exp[(V_m_ − V_1/2_)/*k*]), resulting in V_1/2_ (the potential of half maximal inactivation) and the slope factor *k*.

Steady-state SI of wildtype and mutant hNav1.7 was induced by a 30 s conditioning pulse to various potentials in steps of 20 mV followed by a 100 ms step to −120 mV to remove fast inactivation. Channel availability was assessed by a 40 ms test pulse to 0 mV. To guarantee full recovery of inactivation a 90 s sweep interval was used. To correct for channel rundown during the protocol an additional 10 ms test pulse to 0 mV was implemented before the conditioning pulse and served as reference. The same voltage protocol was used to compare SI of hNav1.7 and mNav1.6r WT seen in Fig. 3.

To examine steady-state SI of mNav1.6 and its mutants a conditioning prepulse of 60 s ranging from −130 to +10 mV in steps of 10 mV was followed by a 100 ms step to −120 mV to remove fast inactivation and a test pulse to +20 mV for 40 ms, at which available channels were assessed. Normalized current amplitude was plotted against prepulse potential and fitted by a Boltzmann equation as described for steady-state fast inactivation.

SI, activation and steady-state fast inactivation were determined in the presence of 500 μM LCM in the external bath solution (preincubation), where indicated. The effect of LCM on resurgent currents was obtained by perfusion of external bath solution containing 500 μM LCM to each recorded cell.

### Chemicals

All chemicals were purchased from Sigma (Germany) or Merck (Germany) unless otherwise stated. A stock solution of the β4-peptide (sequence ‘KKLITFILKKTREK’, PSL GmbH, Heidelberg, Germany) was diluted in internal solution to a final concentration of 100 μM and kept on ice during the process of experiments.

Recombinant ATX-II (*Anemonia sulcata* toxin, Alomone Labs, Jerusalem, Israel or Sigma-Aldrich, USA) was added to the bath solution to a final concentration of 50 nM.

LCM (Bicoll GmbH, Munich) was dissolved in ETOH (500mM) and stored at −20 °C. External bathing solutions with final concentrations were mixed on the day of experiments.

### Data analysis and statistics

Data were analyzed and graphed using Fitmaster software (HEKA Elektronik), Igor Pro 5.2 software (Wavemetrics), Origin 9.2 (OriginLab) and Corel Draw X3–X6 (Corel Corporation). Data are depicted as mean ± SEM.

Conductance data obtained from activation protocols were normalized to the maximum conductance value. Normalized peak inward currents obtained from steady-state fast inactivation and SI protocols were fitted with a single Boltzmann equation.

For fitting of SI the data points at −100 and −80 mV for Nav1.7/V948C mutation were neglected, to assume a single exponential fit for better comparison.

For statistical testing groups were compared by an ANOVA or a Kruskal-Wallis test in case of non-parametric testing followed by a Tukey post-hoc analysis (significance at least p < 0.05 for*, p < 0.005 for** and p < 0.001 for***).

## Additional Information

**How to cite this article**: Hampl, M. *et al*. Sodium channel slow inactivation interferes with open channel block. *Sci. Rep.* **6**, 25974; doi: 10.1038/srep25974 (2016).

## Supplementary Material

Supplementary Information

## Figures and Tables

**Table 1:**
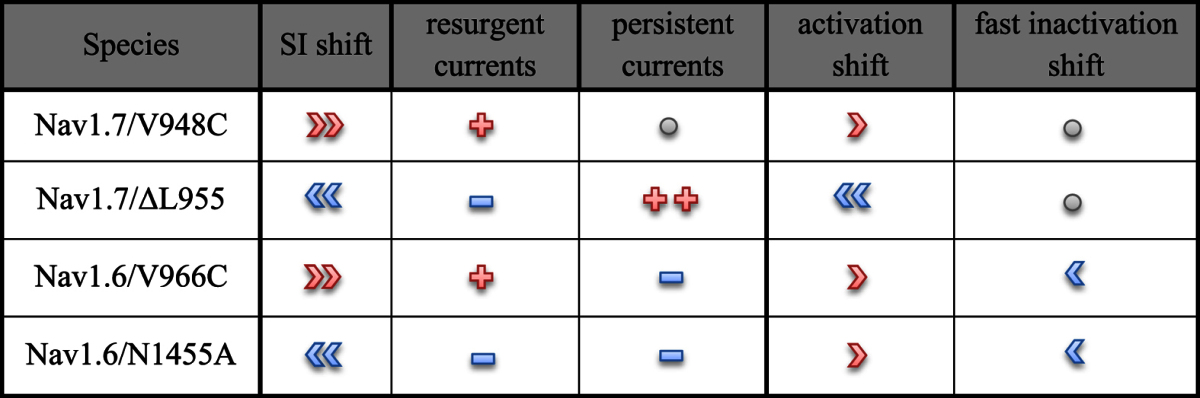
Summary illustration of the presented results.

Illustrated are the depolarized (red right arrow symbols) and hyperpolarized (blue left arrow symbols) voltage-dependencies of activation, fast inactivation and SI. An increase of resurgent or persistent currents is represented by red plus symbols and a decrease by blue minus symbols. The number of symbols stands for the strength of alteration. No changes are shown as grey circles.
