# Supplementary material for: Sodium channel slow inactivation interferes with open channel block
Source: Sci Rep. 2016 May 13;6:25974. doi: 10.1038/srep25974 (PMC4865801; doi:10.1038/srep25974)
Supplement: Supplementary Information [file srep25974-s1.doc]

**Sodium channel slow inactivation interferes with open channel block**

**Martin Hampl*^1,2^, Esther Eberhardt*^1,3^, Andrias O. O’Reilly^1,4^, Angelika Lampert^1,2^**

1 Institute of Physiology and Pathophysiology Friedrich-Alexander Universität Erlangen-Nürnberg, Universitaetsstrasse 17, 91054 Erlangen,

2 Institute of Physiology, RWTH Aachen University, Pauwelsstrasse 30, 52074 Aachen, Germany

3 Department of Anesthesiology, Universitaetsklinikum Erlangen, Friedrich-Alexander-Universität Erlangen-Nürnberg, Krankenhausstrasse 12, 91054 Erlangen

4 School of Natural Sciences and Psychology, Liverpool John Moores University, Liverpool, UK

Running title: Slow inactivation reduces resurgent currents

**SUPPLEMENTS**

**Fig. S1:**

**
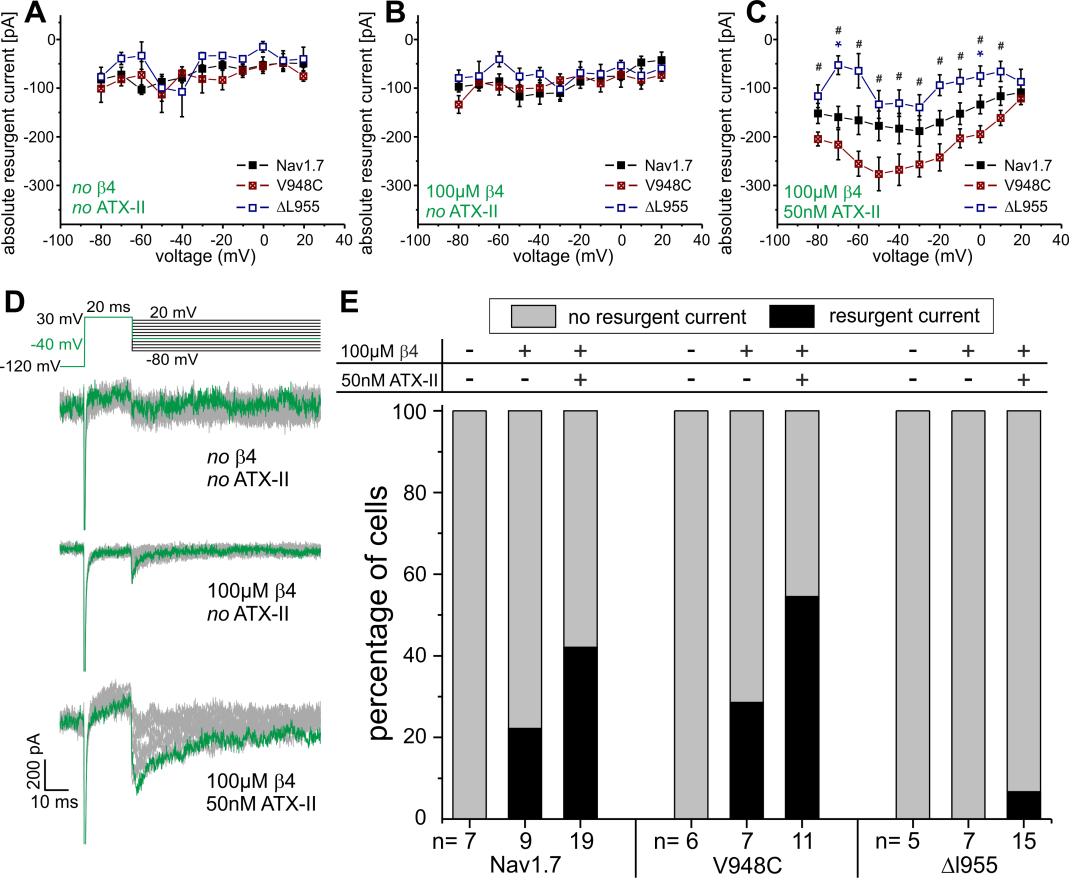
**

**Figure S1:**

*Resurgent currents are observed with the β4 peptide in the internal recordings solution.*

(A-C) Absolute resurgent currents are shown when recorded with neither β4 nor ATX-II present (A, n = 5-7), in the presence of 100 µM β4 in the pipette (B, n = 7-9) or additionally with 50 nM ATX-II in the external recording solution (C, n = 11-19). Only the last condition yielded reliable resurgent currents. (D) Representative resurgent currents traces of Nav1.7 recorded at the indicated conditions. The trace recorded at -40 mV is shown in green.
(C) Resurgent currents are detectable in WT and Nav1.7/V948C, but only in 6.7% of cells transfected with Nav1.7/ΔL955 and only when ATX-II and β4 are present (E). Significant differences between Nav1.7 WT and Nav1.7/ΔL955 are marked with blue asterisks (*) and between Nav1.7/ΔL955 and Nav1.7/V948C with #, p < 0.05.

**Fig.S2:**

**
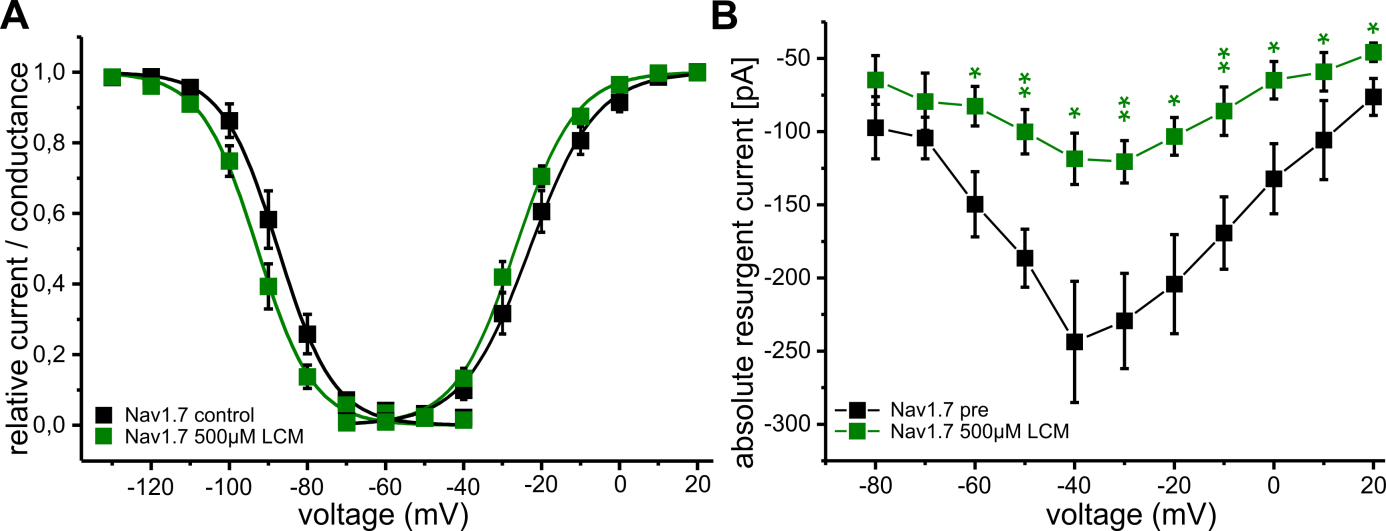
**

**Figure S2:**

*LCM reduces resurgent currents of Nav1.7 WT*

(A) Normalized conductance–voltage relationship of Nav1.7 control (filled black squares, V_1/2_ = -23.0 ± 2.2 mV, n = 6) is not altered by 500 µM LCM (filled green squares, V_1/2_ = -26.8 ± 1.3 mV, n = 6). V_1/2_ of steady-state fast inactivation (filled black squares, V_1/2_ = -87.6 ± 2.2 mV , n = 6) is unchanged by 500 µM LCM (filled green squares, V_1/2_ = -92.7 ± 1.7 mV, n= 6). (B) Absolute resurgent current amplitude (black squares, n = 6) decreased after the application of 500 µM LCM (filled green squares, n=6). Significant differences are marked with green asterisks: * p < 0.05 and ** p < 0.005 (paired t-test).

**Tab.S1:**

**Table S1:**

| Species | activation | | fast inactivation | |
| --- | --- | --- | --- | --- |
|  | V_1/2_ [mV] (n) | slope *k* [mV] (n) | V_1/2_ [mV] (n) | slope *k* [mV] (n) |
| hNav1.7 WT | -26.8 ± 1.6 (6) | 7.0 ± 0.39 (6) | -81.4 ± 1.9 (6) | 6.5 ± 0.3 (6) |
| hNav1.7/V948C | -18.7 ± 1.5 (7) ** | 9.3 ± 0.41 (7) ** | -89.2 ± 3.7 (7) | 5.7 ± 0.3 (7) |
| hNav1.7/ΔL955 | -44.5 ± 1.3 (7) *** | 7.9± 0.45 (7) | -81.2 ± 1.5 (7) | 7.4 ± 0.6 (7) |
| mNav1.6r WT | -25.4 ± 1.2 (12) | 5.4 ± 0.4 (12) | -67.5 ± 1.0 (11) | 6.4 ± 0.1 (11) |
| mNav1.6r/V966C | -15.5 ± 0.7 (14) *** | 7.1 ± 0.2 (14) *** | -70.6 ± 0.8 (16) * | 6.9 ± 0.2 (16) |
| mNav1.6r/N1455A | -8.7 ± 0.6 (12) *** | 8.1 ± 0.2 (12) *** | -76.1 ± 0.5 (12) *** | 6.9 ± 0.2 (12) |

*Results of Boltzmann fits of activation and steady-state fast inactivation of Nav1.7, Nav1.6 and their mutations*

Results are displayed as mean and SEM. The table presents data related to Fig. 1 and Fig. 4 in the main text. (n) represents the number of measured cells. * Statistically significant difference of the mutation compared to the respective WT (Tukey’s test with * P < 0.05, ** P < 0.005, *** P < 0.001).

**Tab.S2:**

| Species (prepulse duration) | slow inactivation | | |
| --- | --- | --- | --- |
|  | V_1/2_ [mV] (n) | slope *k* [mV] | relative block [%] |
| hNav1.7 WT (30 s) | -63.5 ± 2.1 (10) | 12.0 ± 1.2 | 90.0 ± 1.5 |
| hNav1.7/V948C (30 s) | -47.3 ± 2.8 (6) *** | 22.3 ± 2.7 ** | 89.4 ± 2.8 |
| hNav1.7/ΔL955 (30 s) | -105.4 ± 2.5 (6) *** | 5.5 ± 0.6 * | 83.6 ± 2.2 |
| hNav1.7 WT 500 µM LCM (30 s) | -99.8 ± 1.2 (6) *** | 7.4 ± 0.6 ** | 90.2 ± 1.5 |
| mNav1.6r WT (30 s) | 43.4 ± 1.2 (7) *** | 10.2 ± 0.8 | 66.9 ± 3.7 *** |
| mNav1.6r WT (60 s) | -59.2 ± 1.5 (12) | 10.9 ± 0.3 | 75.8 ± 1.1 |
| mNav1.6r/V966C (60 s) | -36.6 ± 5.6 (4) *** | 19.3 ± 1.0 *** | 72.7 ± 2.9 |
| mNav1.6r/N1455A (60 s) | -81.9 ± 2.5 (4) *** | 7.9 ± 0.5 ** | 76.5 ± 2.3 |

**Table S2:**

*Results of slow inactivation of Nav1.7, Nav1.6 and their mutations*

Results are displayed as mean and SEM. (n) represents the number of measured cells. Asterisks signify statistically significant difference to the respective WT. SI results of mNav1.6r WT (30 s) were compared with hNav1.7 WT (30 s) (Tukey’s test with * P < 0.05, ** P < 0.005, *** P < 0.001).
